# Paradoxical effect of minocycline on established neuropathic pain in rat

**DOI:** 10.17179/excli2016-434

**Published:** 2017-03-08

**Authors:** Malek Zarei, Masoumeh Sabetkasaei, Taraneh Moini-Zanjani

**Affiliations:** 1 Department of Pharmacology, Faculty of Medicine, Shahid Beheshti University of Medical Sciences, Tehran, Iran

**Keywords:** minocycline, neuropathic pain, microglia, allodynia, hyperalgesia, rat

## Abstract

Neuropathic pain occurs after peripheral nerve damage, inflammation or infection. In this situation, microglial cells become activated and play a key role in producing pain. Minocycline (microglia inhibitor), was reported to reduce pain when used preventively. However, it seems that, when used after nerve injury, results in its pain reducing effects are different. In this regard, to assess the pain reducing differences of minocycline, neuropathic pain was induced by the ligation of the sciatic nerve in the rat which is recognized as chronic constriction injury (CCI) and minocycline was administered before and after sciatic nerve injury. Wistar male rats (200-250 g, n=6) were used in these experiments. Rats were distributed in various groups: vehicle-treated CCI (control), sham-operated and minocycline-treated CCI groups. In the first part of the experiment (pre-injury study), minocycline (10, 20, 30 and 40 mg/kg,) was injected one hour before surgery and then daily for two weeks. In the second part (post injury study), minocycline was administered: 1: at day one after nerve damage once a day to day 14, 2: at day seven after surgery and continued daily until day 14. Analgesimeter for thermal hyperalgesia and von Frey hairs for mechanical allodynia were used to evaluate pain behavior. Thermal hyperalgesia and mechanical allodynia were attenuated significantly, when minocycline used before surgery, while it was not able to reduce pain behavior administered after surgery. It seems that, in spite of what some previous studies have reported, here, minocycline is not able to attenuate established neuropathic pain.

## Introduction

Neuropathic pain arising from functional changes in the pain sensory systems following damage to the nerve, affects millions of people worldwide (Tsuda et al., 2005[17]). Currently, treatment of this prevalent condition is not satisfactory (Zimmermann, 2001[23]). To date, there is no clear mechanism underlying neuropathic pain. Common analgesic drugs, like anti-inflammatory drugs and opioids, are used to reduce neuropathic pain, but in some patients, they are not effective and require high dosages which can cause unacceptable side effects (Wang et al., 2011[18]).

Microglia are increasingly considered as a crucial factor in the appearance of neuropathic pain after damage to the peripheral nerve (Xiaodi et al., 2010[20]). Microglia marker expression, proliferation and morphological changes were seen following sciatic nerve injury, chronic constriction injury (CCI) and sciatic nerve ligation (SNL) (Echeverry et al., 2008[5]). Minocycline which is considered as an inhibitor of microglial cells, has no effect on neurons and astrocytes (Tikka and Koistinaho, 2001[16]). So far, the effects of minocycline on neuropathic pain have been evaluated in many experiments (Guasti et al., 2009[6]; Ledeboer et al., 2005[8]; Padi and Kulkarni, 2008[12]; Raghavendra et al., 2003[14]; Zanjani et al., 2006[21]). In this situation, some studies have shown that minocycline can exert an anti-allodynic and anti-hyperalgesic effect when administered before nerve injury. Nevertheless, some other reports indicate that minocycline cannot reduce pain behavior after damage to peripheral nerves (Guasti et al., 2009[6]; Padi and Kulkarni, 2008[12]; Raghavendra et al., 2003[14]; Zanjani et al., 2006[21]). On the other hand, some additional studies suggest that minocycline administered post-injury is effective in decreasing pain behavior (Ledeboer et al., 2005[8]; Mei et al., 2011[9]; Nazemi et al., 2012[11]).

As is reported in the literature, it is well recognized that minocycline can decrease neuropathic pain (when administered before nerve damage), yet experiments about such effect after the commencement of nerve damage are controversial. In this regard, in clinical practice, the benefit of using minocycline should logically be after nerve damage. Therefore, this experiment was designed to re-assess the effect of minocycline on the chronic pain model of CCI, when injected before and also after nerve damage.

## Material and Methods

### Animals

In this study, male Wistar rats, aged 3 months (weighing 200-250 g, n = 6) were used. Animals were permitted to accustom to the housing facilities one week before the beginning of the experiments. A quiet room was chosen for the behavioral studies which were performed between the hours of 8:00 and 12:00 AM. The ethical guidelines of the International Association for the Study of Pain (IASP) throughout the experiment were considered (Zimmermann, 1983[22]).

### Surgery

CCI model of neuropathic pain was produced according to Bennett and Xie model (Bennett and Xie, 1988[1]). Ketamine (60 mg/kg) and xylazine (10 mg/kg) were used to anesthetize the animals. After exposing, the left sciatic nerve was tied loosely with four ligatures by chromic cat gut 4-0 thread around the nerve close to the trifurcation with 1 mm space. After irrigation with normal saline, the wound was closed in layers using 4-0 silk thread and surgical skin staples. Rats in sham-operated group experienced surgical procedure, but the ligation was not tied.

### Drug preparation

Saline solution (0.9 % NaCl) was used as a solvent for minocycline (Sigma, USA). The animals were anesthetized with Ketamine/ Xylazine mixture (60/10 mg/kg) injected intraperitoneally (i.p.).

### Drug administration

In this study, two main groups of rats were designed as pre-surgery and post-surgery groups. Each main group was divided into three subgroups: I) vehicle-treated CCI (control) group, II) sham-operated (sham) group and III) minocycline-treated CCI (minocycline) group. Control and Sham animals received saline as a vehicle. In the preventive study, animals received minocycline (10, 20, 30, and 40 mg/kg) 1 h before surgery and continued daily until day 14 post-surgery (Raghavendra et al., 2003[14]). In the post-surgery study, Group 1: rats received minocycline (10, 20, 30, and 40 mg/kg) at day1 post-surgery and continued daily to day 14. Group 2: rats received minocycline (10, 20, 30, and 40 mg/kg) at day 7 post-surgery and continued daily to day 14. In the preventive study, all behavioral tests performed on day 0 which was considered as a control day and continued on days 1, 3, 5, 7, 10, and 14 after nerve ligation. In the post-surgery study, in group 1, behavioral tests were performed at days 0, 1, 3, 5, 7, 10 and 14, and in group 2, behavioral tests were done at days 0, 7, 10, and 14 after nerve damage.

### Behavioral tests and experimental design

Noxious and innocuous stimuli were used for evaluating of the sciatic nerve sensitivity. Animals were placed into the chambers 30 min before testing to accustomed to the testing chambers. Hyperalgesia and allodynia were evaluated in animals. The order of behavioral tests was thermal hyperalgesia and mechanical allodynia, 30 min was considered between each assay.

### Thermal hyperalgesia

Hargreaves test was used for the assessment of thermal hyperalgesia (Hargreaves et al., 1988[7]). A beam of radiant heat was focused on the plantar surface of the left paw through a transparent Perspex surface (Ugo Basile, Comerio, Italy). The withdrawal latency was recorded with a cut-off time of 20 s to prevent tissue damage. The left paw was tested five times, and 5 min was considered as an interval between each test. The average value of the withdrawal latency of five consecutive tests was recorded.

### Mechanical allodynia

A set of calibrated nylon monofilaments (Stoelting, USA) was applied to measure mechanical sensitivity to non-noxious stimuli. For the assessment of mechanical sensitivity, von Frey test was performed (Chaplan et al., 1994[2]). A transparent plexiglass cage on an elevated metal screen surface with 1 cm mesh openings was used. The left hind paw of each animal was exposed to the increasing intensity of von Frey filaments sequentially. The paw withdrawal threshold was considered as the lowest von Frey stimulus eliciting two sequential responses with 3 min intervals between them (withdrawal from pressure). Increasing stimuli from 2 to 60 g were delivered to the plantar surface of the paw using successively greater diameter filaments until the hind paw was withdrawn. In the absence of a response, to avoid tissue damage, 60 g was considered as the cut-off point. 

### Statistical analysis

One-way repeated measures analysis of variance (RM-ANOVA), followed by a post-hoc Tukey's test was used to analyze the parametric data. Related samples followed by the Wilcoxon test was used to analyze the non-parametric data. In all cases, data expressed as mean ± SEM (standard error) and P-values less than 0.05 were considered significant. Data were analyzed using the statistical package “SPSS 18”.

## Results

### Effect of preventive administration of minocycline on pain behaviors in thermal hyperalgesia test 

**Figure 1:**
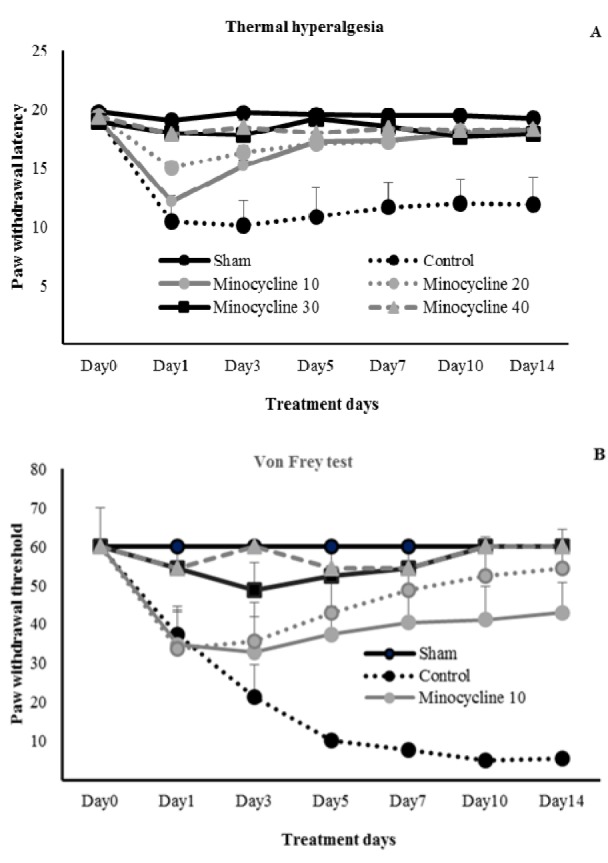
Effect of pre-injury administration of minocycline in various treatment days on thermal hyperalgesia and mechanical allodynia. Minocycline (10, 20, 30 and 40 mg/kg; i.p.) from day 0 before nerve injury to day 14 was injected, and paw withdrawal latency (A) and paw withdrawal threshold (B) were measured. Data were shown as mean ± S.D.

Minocycline (10, 20, 30, and 40 mg/kg) decreased thermal hypersensitivity in the drug-treated CCI group during the period of study when compared to day 0. Minocycline 10 and 20 mg/kg decreased pain in days 10 and 14, while with the dosages of 30 and 40 mg/kg a decrease in pain behavior was started at day one and this effect was continued until day 14 compared to day 0. A significant decrease in pain behavior was found by minocycline 10 mg/kg in comparison to vehicle-treated CCI animals (*P*<0.001) which was initiated from day 3 and continued to day 14. On the other hand, in comparison to vehicle-treated CCI group, minocycline 20, 30, and 40 mg/kg alleviated the pain behavior one day after the nerve damage which was continued throughout the study period. Vehicle-treated CCI group showed thermal hyperalgesia (P<0.01) at the first day following nerve injury, which was sustained throughout the experimental period. Sham-operated rats did not show pain behavior during the 14 days of the study compared to the control day (Figure 1A(Fig. 1)). 

In mechanical allodynia test, vehicle-treated CCI group showed allodynia one day following nerve ligation (*P*<0.05) compared to day 0, this effect continued until the end of the study. Compared to day 0, minocycline with the different dosages controlled the allodynic pain. The pain reducing effects of minocycline 30 and 40 mg/kg was seen one day after nerve ligation while these effects, with dosages of 10 and 20 mg/kg were appeared at days 7 and 5 following nerve injury respectively. Furthermore, minocycline (10, 20, 30, and 40 mg/kg) showed anti-allodynic effects in comparison to vehicle-treated CCI rats (*P*<0.001). Sham-treated animals did not produce allodynic pain compared to day 0 before surgery (Figure 1B(Fig. 1)). 

### Effect of post-injury administration of minocycline on pain behaviors

**Figure 2:**
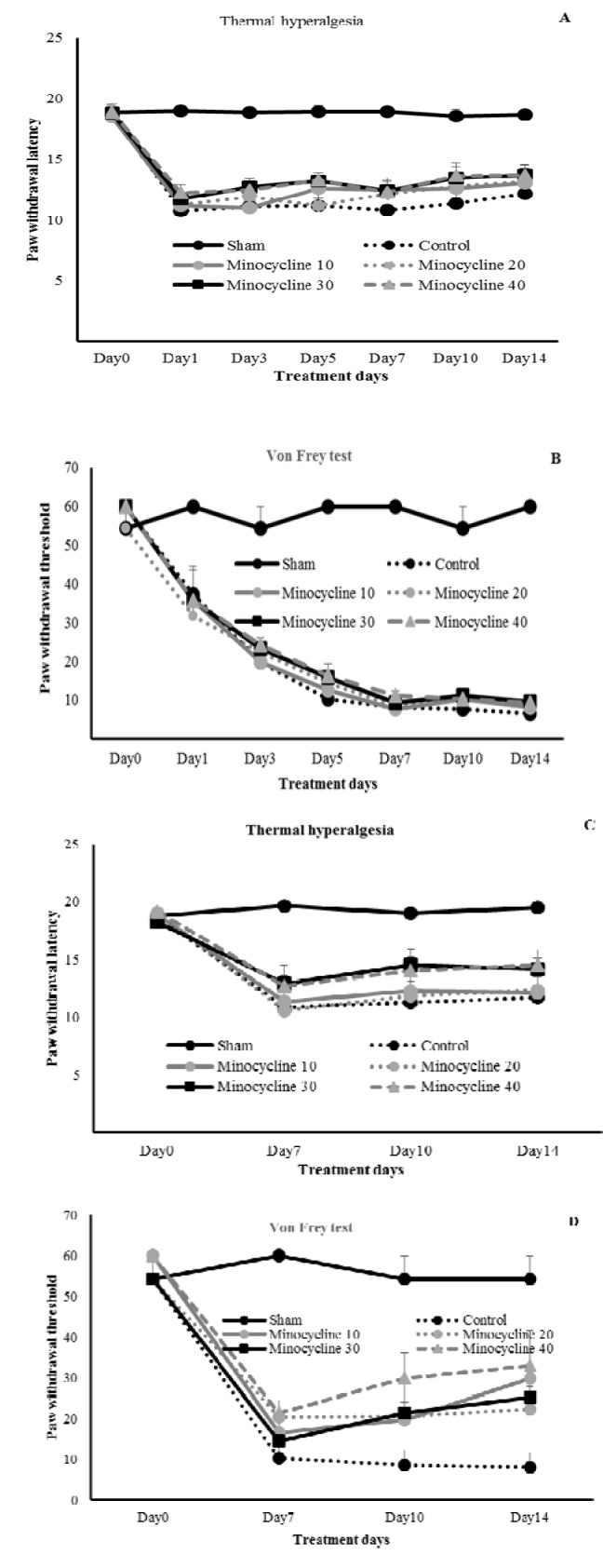
Effect of minocycline on established thermal hyperalgesia and mechanical allodynia in various treatment days. Minocycline (10, 20, 30 and 40 mg/kg; i.p.) was injected at day 1 and 7 and continued until day 14 respectively. Paw withdrawal latency in response to the beam of radiant heat (A, C) and paw withdrawal threshold in response to Von Frey filaments (B, D) were assessed. Data were presented as mean ± S.D.

Group 1: Daily administration of minocycline (10, 20, 30, and 40 mg/kg) from day one to day 14 in the post-injury model, had no effects in decreasing pain behaviors in response to thermal and mechanical stimuli respectively (Figure 2A(Fig. 2), 2B(Fig. 2)). 

Group 2: Daily administration of minocycline (10, 20, 30, and 40 mg/kg) at day seven to day 14 in the post-injury model, had no effects in reducing pain behaviors in response to thermal and mechanical stimuli respectively (Figure 2C(Fig. 2), 2D(Fig. 2)).

See more results in Supplementary Data.

## Discussion

In this study, the effects of minocycline on neuropathic pain before and after nerve damage were assessed. The experiment was performed in two ways: pre and post-injury studies. We found that administration of minocycline before nerve injury attenuated pain sensitivity, while its use following nerve damage did not reduce already existing pain.

To date, to create a model simulating neuropathic pain in a human, various studies have been performed with a focus on damage to the sciatic nerve, in this regard, CCI is one of the models, which can produce significant pain in animals (Mika et al., 2007[10]). Data from the studies on chronic pain show an abnormal excitability of the neurons in neuropathic pain. Following peripheral nerve injury in primary sensory ganglia and the dorsal horn of the spinal cord some functional and anatomical alterations of neurons have been detected (Costigan et al., 2009[4]). Apart from neurons, there are many data indicating these changes also arise in glial cells, particularly microglia (Scholz and Woolf, 2007[15]). It is most likely that when neuropathic pain is produced by CCI, activation of spinal microglia plays the main role in this process. Moreover, neuropathic pain can be controlled efficiently when these cells are inhibited (Mika et al., 2007[10]; Raghavendra et al., 2003[14]; Tsuda et al., 2005[17]). It seems that there is a relationship between microglia activation, proinflammatory cytokines and pain signaling pathways (Watkins and Maier, 2003[19]). Furthermore, studies demonstrate that minocycline can protect neurons against traumatic brain injury and neurodegenerative diseases (Choi et al., 2007[3]). These effects strongly show that microglia activation is inhibited in these situations. 

Studies on minocycline mechanism of action show that it inhibits MAP kinase pathway in microglia and can decrease their activities (Piao et al., 2006[13]). Increasingly, experiments indicate that minocycline can attenuate pain when used before nerve injury (Guasti et al., 2009[6]; Padi and Kulkarni, 2008[12]; Raghavendra et al., 2003[14]; Zanjani et al., 2006[21]). Although the majority of evidence emphasize on the effectiveness of this drug in reducing neuropathic pain injected before nerve damage, nevertheless the results are strongly contradictory when used at various times after nerve injury. It has been shown that, during a period of 14 days experimental studies, minocycline decreases pain hypersensitivity when administered one hour before nerve injury (Guasti et al., 2009[6]; Zanjani et al., 2006[21]). In another study, data show that minocycline relieves pain during ten days of the study when injected preventively (Raghavendra et al., 2003[14]). In a 28 days experiment, it has also been shown, that, repeated administration of minocycline initiated 2 hours before nerve damage inhibits the development of mechanical hypersensitivity until day 21 (Padi and Kulkarni, 2008[12]). Similarly, we obtained same results concerning pain reducing effects of minocycline used preventively.

However, there are different aspects of the effects of minocycline on neuropathic pain when administered following damage to neurons or in already established pain. Many data indicate that minocycline cannot relieve the existing pain (Padi and Kulkarni, 2008[12]; Raghavendra et al., 2003[14]; Zanjani et al., 2006[21]). In this regard, one study shows that i.p. administration of minocycline started at day five post-ligation until day 10, has not been able to attenuate existing hyperalgesia and allodynia (Raghavendra et al., 2003[14]). Another study reports that a single injection of minocycline at day four throughout the progression of pain hypersensitivity has no effect on mechanical stimulation in the CCI model. This experiment has also indicated that minocycline, when used daily, from day 7 to day 28 post-ligation cannot decrease mechanical stimulation during the period of the study (Padi and Kulkarni, 2008[12]). In the present study, our data showed that, injection of minocycline at day 1 (continued daily to day 14) and at day 7 (continued daily to day 14) after nerve damage, could not reduce allodynia and hyperalgesia which is in accordance with the results of some previous studies on minocycline administered after nerve damage.

On the other hand, contrary to what mentioned above, there are some other reports indicating that, minocycline can decrease pain when administered following damage to the nerve. It has been shown that a single dose of minocycline used post-nerve ligation, completely reverses pain behavior induced by SNL, in which the drug alleviates mechanical allodynia only at days 1, 3 and 7 and not on days 10 or 21 following nerve lesion (Mei et al., 2011[9]). Another research indicates that chronic injection of minocycline for eight days initiated from day six following nerve ligation, the drug attenuates allodynia and hyperalgesia at day 13 postoperatively (Nazemi et al., 2012[11]). Moreover, it has been shown that minocycline is able to attenuate the allodynia one day, but not one week following nerve ligation (Ledeboer et al., 2005[8]).

In conclusion, based on the analgesic effects of minocycline used pre and post nerve injury model and what we found in this regard, it seems that there are some differences in minocycline action in relieving neuropathic pain which is needed more complementary studies to obtain a clear explanation in this field. In fact, as it was reported in the previous studies, our data supported the preventive use of minocycline in reducing pain hypersensitivity behavior, but it failed to inhibit pain when used after an injury to the sciatic nerve in CCI model. However, we suggest that the evaluation of minocycline effects at different time points, dosages in different nerve injury models of neuropathic pain needs to be done after nerve damage to attain a comprehensive result for its possible use in already established chronic pain in the clinic. 

## Acknowledgements

This study is extracted from Malek Zarei's Ph.D. Dissertation of Pharmacology, Shahid Beheshti University of Medical Sciences, Tehran, Iran.

## Conflict of interest

The authors have no conflict of interests.

## Supplementary Material

Supplementary data
