# Supplementary material for: Paradoxical effect of minocycline on established neuropathic pain in rat
Source: EXCLI J. 2017 Mar 8;16:229–35. doi: 10.17179/excli2016-434 (PMC5427462; doi:10.17179/excli2016-434)
Supplement: Supplementary data [file EXCLI-16-229-s-001.pdf]

EXCLI Journal 2017;16:229-235 – ISSN 1611-2156
Received: June 06, 2016, accepted: February 09, 2017, published: March 08, 2017

Supplementary data to:

PARADOXICAL EFFECT OF MINOCYCLINE ON ESTABLISHED NEUROPATHIC PAIN IN RAT

Malek Zarei[1], Masoumeh Sabetkasaei[1], Taraneh Moini-Zanjani[1*]

[1] Department of Pharmacology, Faculty of Medicine, Shahid Beheshti University of Medical Sciences, Tehran, Iran

* Taraneh Moini-Zanjani, Ph.D, Assistant Professor, Department of Pharmacology, Faculty of Medicine, Shahid Beheshti University of Medical Sciences, Evin, Daneshjou Blvd, Koudakyar Ave, Tehran, Iran. Tel: +982122439969, Fax: +982122439969, P.O.BOX: 19395-4719, Postcode: 19857-17443
E-mail: tzanjani@yahoo.com, t.moini@sbmu.ac.ir

http://dx.doi.org/10.17179/excli2016-434

This is an Open Access article distributed under the terms of the Creative Commons Attribution License (http://creativecommons.org/licenses/by/4.0/).

Table 1: Data obtained from Paw withdrawal threshold in response to Von Frey filaments in different days and various groups when minocycline injected before nerve injury.

Mechanical Allodynia							
**Day**	0	1	3	5	7	10	14
**Sham**	60	60	60	60	60	60	60
**Control**	60	37.33 ± 7.16	21.50 ± 8.14	10.17 ± 1.04	7.67 ± 80	5.00 ± .68	5.67 ± .95
**M10**	60	34.66 ± 8.40	32.83 ± 8.96	37.33 ± 7.16	40.33 ± 9.11	41.17 ± 8.58	43 ± 7.6
**M20**	60	33.67 ± 8.5	35.50 ± 7.94	43.00 ± 7.6	48.67 ± 7.16	52.50 ± 7.5	54.33 ± 5.66
**M30**	60	54.33 ± 5.66	48.67 ± 7.16	52.50 ± 7.5	54.33 ± 5.66	60	60
**M40**	60	54.33 ± 5.66	60	54.33 ± 5.66	54.33 ± 5.66	60	60

M10= Minocycline 10 mg/kg
M20= Minocycline 20 mg/kg
M30= Minocycline 30 mg/kg
M40= Minocycline 40mg/kg.
Data were presented as mean ± S.D.

EXCLI Journal 2017;16:229-235 – ISSN 1611-2156
Received: June 06, 2016, accepted: February 09, 2017, published: March 08, 2017

Table 2: Data obtained from Paw withdrawal latency in response to radiant heat in different days and various groups when minocycline injected before nerve injury.

Thermal Hyperalgesia - pre							
**Day**	0	1	3	5	7	10	14
**Sham**	19.72 ± .09	18.98 ± .43	19.66 ± .15	19.49 ± .3	19.39 ± .29	19.42 ± .23	19.14 ± .49
**Control**	19.08 ± .54	10.45 ± 1.41	10.05 ± 2.14	10.83 ± 2.43	11.65 ± 2.08	11.96 ± 2.02	11.89 ± 2.26
**M10**	19.11 ± .10	12.14 ± .52	15.15 ± .50	17.17 ± .36	17.31 ± .55	17.88 ± .53	18.27 ± .37
**M20**	19.36 ± .18	14.98 ± .43	16.34 ± .54	17.08 ± .67	17.25 ± .64	18.12 ± .68	17.91 ± .53
**M30**	18.97 ± .29	17.92 ± .63	17.78 ± .33	19.19 ± .20	18.54 ± .53	17.60 ± 1.19	17.85 ± .37
**M40**	19.37 ± .25	17.88 ± .50	18.45 ± .54	17.92 ± .63	18.39 ± .51	18.17 ± .56	18.30 ± .35

M10= Minocycline 10 mg/kg
M20= Minocycline 20 mg/kg
M30= Minocycline 30 mg/kg
M40= Minocycline 40mg/kg.
Data were presented as mean ± S.D.

Table 3: Data obtained from Paw withdrawal threshold in response to Von Frey filaments in different days and various groups when minocycline administered after nerve injury.

Mechanical Allodynia							
**Day**	0	1	3	5	7	10	14
**Sham**	54.33 ± 5.67	60	54.33 ± 5.67	60	60	54.33 ± 5.67	60
**Control**	60	37.33 ± 7.17	19.67 ± 2.93	10.17 ± 1.05	8 ± .73	7.67 ± .80	6.33 ± .61
**M10**	60	35.50 ± 7.94	19.67 ± 2.93	12.50 ± 1.12	7.67± .80	10.17 ± 1.05	8.00 ± .73
**M20**	54.33 ± 5.67	31.66 ± 5.66	22.33 ± 2.32	14.33 ± 2.54	8.33 ± .61	11.00 ± 1.32	8.33 ± .80
**M30**	60	35.50 ± 7.94	23.33 ± 2.67	15.83 ± 3.35	9.17 ± 1.28	11.00 ± 1.32	9.50 ± 1.26
**M40**	60	35.50 ± 7.94	24.17 ± 1.83	16.17 ± 3.21	11.00 ± 1.32	10.17 ± 1.05	9.17 ± 1.38

M10= Minocycline 10 mg/kg
M20= Minocycline 20 mg/kg
M30= Minocycline 30 mg/kg
M40= Minocycline 40mg/kg.
Data were presented as mean ± S.D.

EXCLI Journal 2017;16:229-235 – ISSN 1611-2156
Received: June 06, 2016, accepted: February 09, 2017, published: March 08, 2017

Table 4: Data obtained from Paw withdrawal threshold in response to Von Frey filaments in different days and various groups when minocycline injected after nerve injury.

Mechanical Allodynia - post				
**Day**	0	7	10	14
**Sham**	54.33 ± 5.67	60	54.33 ± 5.67	54.33 ± 5.67
**Control**	54.33 ± 5.67	10.33 ± 3.20	8.67 ± 3.49	8.00 ± 3.61
**M10**	60	16.67 ± 3.16	19.67 ± 2.93	29.83 ± 6.30
**M20**	54.33 ± 5.67	20.50 ± 2.46	20.67 ± 3.37	22.33 ± 5.67
**M30**	54.33 ± 5.67	14.50 ± 2.74	21.50 ± 8.15	25.33 ± 7.42
**M40**	60	21.50 ± 2.91	29.83 ± 6.29	32.83 ± 8.96072

M10= Minocycline 10 mg/kg
M20= Minocycline 20 mg/kg
M30= Minocycline 30 mg/kg
M40= Minocycline 40mg/kg.
Data were presented as mean ± S.D.

Table 5: Data obtained from Paw withdrawal latency in response to radiant heat in different days and various groups when minocycline administered after nerve injury.

Thermal Hyperalgesia - post							
**Day**	0	1	3	5	7	10	14
**Sham**	18.88 ± .32	19 ± .40	18.83 ± .37	18.94 ± .47	18.90 ± .35	18.57 ± .49	18.68 ± .32
**Control**	18.89 ± .29	10.73 ± .50	11.13 ± .55	11.18 ± 1.08	10.80 ± .4	11.37 ± 1.37	12.15 ± .88
**M10**	18.51 ± .36	11.18 ± .38	11.02 ± .76	12.60 ± .65	12.46 ± .79	12.59 ± 1.22	13.02 ± 1
**M20**	19.11 ± .44	11.23 ± .86	11.93 ± 1	11.21 ± .61	12.17 ± .54	12.77 ± .96	13.13 ± 1.13
**M30**	18.80 ± .22	11.71 ± .55	12.69 ± .44	13.18 ± .7	12.40 ± 1.03	13.46 ± 1.21	13.67 ± .83
**M40**	18.88 ± .29	12.21 ± .7	12.49 ± .93	13.24 ± .37	12.28 ± .85	13.62 ± .78	13.69 ± .87

M10= Minocycline 10 mg/kg
M20= Minocycline 20 mg/kg
M30= Minocycline 30 mg/kg
M40= Minocycline 40mg/kg.
Data were presented as mean ± S.D.

EXCLI Journal 2017;16:229-235 – ISSN 1611-2156
Received: June 06, 2016, accepted: February 09, 2017, published: March 08, 2017

Table 6: Data obtained from Paw withdrawal latency in response to radiant heat in different days and various groups when minocycline administered after nerve injury.

Thermal Hyperalgesia - post				
**Day**	0	7	10	14
**Sham**	18.77 ± .17	19.66 ± .15	18.98 ± .44	19.49 ± .31
**Control**	18.73 ± .53	10.85 ± 1.76	11.27 ± 1.85	11.73 ± 1.71
**M10**	18.85 ± .31	11.35 ± .71	12.25 ± 1.22	12.14 ± 1.14
**M20**	18.97 ± .46	10.58± .53	11.80 ± .86	12.36 ± 1.25
**M30**	18.23 ± .21	12.92 ± 1.61	14.48 ± 1.41	14.17 ± 1.65
**M40**	19.17 ± .4	12.68 ± .91	14.02 ± 1.17	14.52 ± .57

M10= Minocycline 10 mg/kg
M20= Minocycline 20 mg/kg
M30= Minocycline 30 mg/kg
M40= Minocycline 40mg/kg.
Data were presented as mean ± S.D.
